# A study of apolipoprotein A1(ApoA1) and interleukin-10(IL-10) in diabetes with foot ulcers

**DOI:** 10.37796/2211-8039.1279

**Published:** 2022-03-01

**Authors:** Rachita Nanda, Suprava Patel, Amritava Ghosh, K.S. Asha, Eli Mohapatra

**Affiliations:** a Department of Biochemistry, All India Institute of Medical Sciences (AIIMS), Raipur, Chhattisgarh, 492099, India; b Department of Endocrinology, All India Institute of Medical Sciences (AIIMS), Raipur, Chhattisgarh, 492099, India

**Keywords:** ApoA1, Cytokines, Diabetic foot ulcers, eGFR, TNF-α, Wagners

## Abstract

**Background:**

The high morbidity, mortality and associated economic burden have entailed to identifying early biomarker of diabetic foot ulcers (DFU). Pro-inflammatory and anti-inflammatory molecules play a role in the chronic inflammation associated with diabetic foot ulcers (DFU).

**Aim:**

This study aims to find the association between ApoA1, IL-10, TNF-α and diabetic foot ulcers, and whether their levels can assess the severity of the disease.

**Method:**

Two groups, diabetic mellitus without foot ulcers and diabetes with foot ulcers were recruited for the study. Detailed clinical history was obtained and blood was collected to measure TNF-α , IL-10 and Apo A1. The association between variables was analysed using Pearson correlation test. ROC analysis was used to identify cut-off values of ApoA1, IL-10 and TNF-α in diabetes patients with foot ulcers.

**Results:**

The presence of pro-inflammatory parameter, TNF-α , was higher and anti-inflammatory biomarkers, HDL, ApoA1 and IL-10 were lower in patients of DFU than those without foot ulcers (p < 0.001). Increasing age, smoking, retinopathy, eGFR and inflammatory biomarkers like low levels of ApoA1 (p < 0.005) and IL-10 (p < 0.001) significantly contributed to the development of diabetic foot ulcers. ROC curve identified the cut-off for ApoA1 and IL-10 as 89.82mg/dL and 78.80pg/mL respectively.

**Conclusion:**

In the light of this study, ApoA1 has the potential to predict DFU. The finding proposes IL-10 (b = −0.37, p < 0.001) could be considered in stratifying DFU as per its severity.

## 1. Introduction

Diabetic foot is associated with high morbidity, mortality, and economic burden [1–4]. The non-healing nature of these ulcers is due to the impairment in usual physiological factors of wound healing which are blocked due to chronic inflammation resulting in the derailment of pro-inflammatory and anti-inflammatory cytokines.

TNF-α, along with associated hyperglycemia, stimulates apoptosis of fibroblasts, keratinocytes, and endothelial cells and accounts for the catabolic state of non-healing ulcers [5,6]. The state of hypoxia in DFU also up-regulates the regular expression of inflammatory cytokines like TNF-α [7]. Thus hyperglycemia and hypoxia have a synergistic effect on the production of pro-inflammatory cytokine release [7]. IL-10, an anti-inflammatory cytokine, inhibits antigen presentation by dendritic cells and macrophage activation and infiltration into the site of injury resulting in inhibition of release of pro-inflammatory cytokines [8]. Low IL-10 has also been observed in wound margins of non-healing ulcers [8,9]. Apo A1 has an anti-inflammatory role by interfering with T-cell signaling of monocytes, hampering the production of IL-1β, TNF-α [10] and blocking the interplay between T cells and monocytes. ApoA1 inflammatory functions of monocyte in peripheral blood mononuclear cells without affecting cell proliferation. In wound healing, ApoA1 interferes with the polymerization of C9 and incorporation in the membrane, thus inhibiting the formation of membrane attack complex of complement [11]. Further, it also increases pentraxin3 to promote efficient tissue repair [12].

This study aims to find the association between ApoA1, IL-10, TNF-α, and diabetic foot ulcers and whether their levels can assess the severity of the disease.

## 2. Materials and methods

### 2.1. Study participants

Seventy-seven participants with type 2 diabetes mellitus (T2DM) and diabetic neuropathy (defined as **Group I)**and 75 participants with T2DM and diabetic neuropathy with foot ulcers (defined as **Group II)** were recruited for this hospital-based observational study by consecutive recruitment.

Patients were recruited from the outpatient clinics of Departments of Endocrinology and Surgery between January to December 2019. The study followed the Declaration of Helsinki guidelines. Institutional ethics committee approval was obtained before initiating research work, and written informed consent was obtained from all participants.

Diabetic neuropathy was defined as loss of protective sensation on testing with 10-g monofilament along with any one of the following: impaired vibration sensation on testing with 128 Hz tuning fork, pinprick sensation, vibration perception threshold, or ankle reflexes. Diabetic foot ulcer (DFU)was defined as patients of diabetes with ‘ulceration, infection, or destruction of deep tissues located in the lower limb below the ankles [13]. The diabetic foot ulcers were classified according to Wagner’s classification [14] [Supplementary Table 1]. Patients with ulcers on both feet or with acute inflammation, liver disease, cardiac failure, varicose veins, peripheral artery disease, malignancy, and psychiatric disorders were not included in the study. All patients were subjected to clinical evaluation and detailed history was recorded with the help of a questionnaire. Data on age, sex, anthropometric measurements, duration of diabetes, and other medical history were obtained. Systolic blood pressure ≥140 mm Hg and diastolic blood pressure ≥90 mm Hg, cholesterol ≥200 mg/dL, and triglycerides ≥150mg/dL were defined as hypertension, hypercholesterolemia, and hypertriglyceridemia, respectively [15,16]. Estimated glomerular filtration rate (eGFR) was calculated using Chronic Kidney Disease Epidemiology Collaboration (CKD-EPI) equation; eGFR < 60 mL/min/1.73 m^2^ was defined as chronic kidney disease (CKD). Blood sample for plasma was collected under aseptic conditions for measurement of ApoA1, IL-10, and TNF-α. ApoA1 was determined in Beckman AU680 analyzer by immunoturbidimetric immunoassay method using commercially available kits from Randox Laboratories, Ltd (UK). The assay range for ApoA1 was 5.78–234 mg/dL, intra assay and inter-assay co-efficient of variation (CV) were 3.08% and 2.04%, respectively. IL-10 and TNF-α levels were measured by enzyme-linked immunosorbent assay (ELISA) method using kits from KRISHGEN Biosystems. The intra and inter-assay CV for TNF-αwere 3.3% and 9%, respectively, and for IL-10 were 12% and 10%, respectively. HbA1c was measured using the HPLC method using the D-10 testing system (Bio-Rad), urea by urease method, creatinine and uric acid by modified Jaffe’s and uricase method, respectively. The cholesterol, triglycerides, and HDL were measured by cholesterol oxidase peroxidase, GPO-PAP, and direct estimation, respectively. LDL was calculated using Friedwald’s equation.

### 2.2. Statistical analysis

The results were statistically analyzed using SPSS 20.0 for Windows and Microsoft Excel. The Kolmogorov–Smirnov test was used to evaluate the normality of variables. Qualitative data were presented as n (%), normally distributed quantitative data as mean ± standard deviation (SD). Data not normally distributed were logarithmically transformed. The difference between groups was compared using an independent sample t-test. Association between variables was analyzed using the Pearson correlation test by measuring the correlation coefficient(r). ROC analysis was used to identify cut-off values of Apo A1, IL-10, and TNF-α in patients with DFU. Multinomial logistic regression analysis was used to identify which independent variables could predict the dependent variables. Results were considered significant when the p-value was <0.05.

## 3. Results

The demographic, clinical, and biochemical characteristics of the two groups are depicted in Table 1. The mean age of patients (54.85 ± 9.6), percentage of males (78.7%), and body mass index (BMI) were higher in Group II than in Group I (p = 0.03, 0.02, and 0.01 respectively). The mean duration of diabetes was more in patients with foot ulcers than those without foot ulcers (p = 0.005) DFU patients had more individuals (42.7%) with the duration of diabetes more than ten years in comparison to those without ulcers (27.2%) (p = 0.04). Only seven patients were on insulin therapy in Group I in comparison to 29 subjects in Group II (p = 0.000). There was a significantly higher number of smokers and individuals with a family history of diabetes in the foot ulcer group than without ulcers (p = 0.03). Amongst the biochemical parameters, glycated hemoglobin A1c (HbA1c), creatinine, and uric acid were significantly higher in Group-II (9.44 ± 1.76, 1.23 ± 0.44, 6.82 ± 2.25 respectively) than in Group-I (8.68 ± 1.86, 1.01 ± 0.23, 5.70 ± 1.37 respectively). The eGFR in patients with ulcers was significantly lower than those without ulcers (p = 0.00). The total cholesterol, triglycerides, and low-density lipoprotein (LDL) were significantly higher in the ulcers group than without ulcers, with lower HDL in Group-II(p = 0.002). Comparing the immunological makers in both groups, subjects in the DFU group showed a lower ApoA1 and IL-10 but higher TNF-α when compared to patients with diabetes without foot ulcers (Group- I). According to Wagner’s classification, there were 2.67% in grade1, 13.3% in grade 2, 38.6% in grade 3, 33.3% in grade 4 and 12% in grade 5.

To identify the association of independent variables in the study population, known risk factors for diabetic foot ulcers were considered for analysis. A significantly higher number of patients with foot ulcers had retinopathy (38.7%) and CKD (44%) than those without ulcers. Patients with diabetes who were obese (BMI > 25 kg/m^2^), smokers, had CKD and had a family history of diabetes had a higher odds ratio. Similarly, total cholesterol > 200 mg/dL (OR = 6.82, 95% CI = 3.25–14.3) and triglycerides > 150 mg/dL (OR = 9.37, 95% CI = 2.06–42.6) was strongly seen in DFU patients. Although there were more hypertensives in the T2DM(n = 31) group than in DFU(n = 20) group, the difference was not statistically significant (p = 0.07) [Table 2].

There was a significant negative and positive correlation of ApoA1 with TNF-α(r = −0.186, p = 0.018) and IL-10 (r = 0.310, p = 0.000), respectively. Similarly, TNF-α and IL-10 (r = −0.224, p = 0.004) were negatively associated with each other [Supplementary Table 2].

Multiple logistic regression analysis as observed by Chi-square and regression coefficients identified increasing age (p = 0.027), smoking (p = 0.032), retinopathy (p = 0.013), eGFR (p = 0.024), HbA1C (p = 0.030) and inflammatory biomarkers like low levels of ApoA1 (p = 0.005) and IL-10 (p = 0.000) to be significantly associated with diabetic foot ulcers [Table 3]. The other variables, gender, BMI, duration of diabetes, hypertension, and TNF-α, were not significant in this model for the study population.

**Fig. 1:**
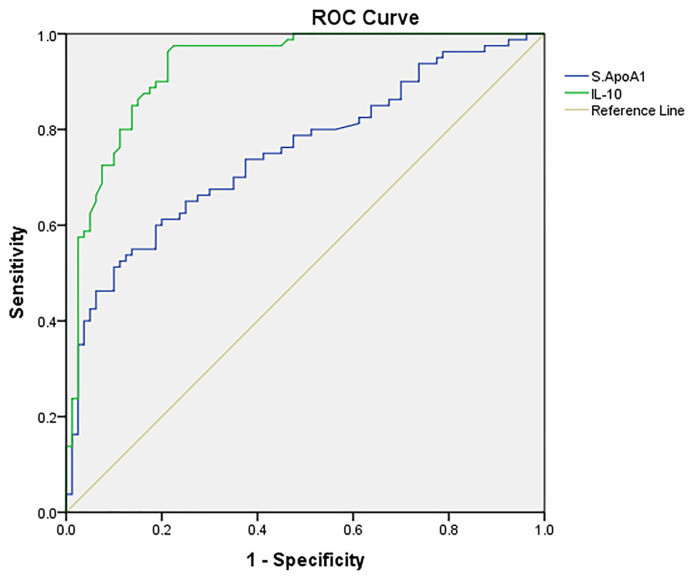
Receiver operator characteristics (ROC) curve of APO A1 and IL-10 in predicting the development foot ulcers in diabetes.

Since the probabilities of inflammatory biomarkers ApoA1 and IL-10 were significant in predicting the presence of DFU, ROC curve analysis was conducted on these to identify the cut-off point for DFU development [Fig. 1]. The cut-offs for ApoA1 and IL-10 were 89.82 mg/dL and 78.80 pg/mL respectively. The sensitivity, specificity, PPV, and NPV of IL-10 were better than ApoA1, as evident by the overall accuracy of 89.5% for IL-10 compared to 75% for ApoA1 [Table 4].

To illustrate whether Apo A1 and IL-10 could predict the severity of ulcers according to their grade, the DFU group was graded to DFU1(grades 0–2) (n = 20) and DFU2(grades 3–5) (n = 55), and tests of difference were utilized. There was no difference in Apo A1 (p = 0.540) and IL-10 (p = 0.856) levels between these two groups [Supplementary Fig. 1].

## 4. Discussion

This study evaluated the association between Apo A1 and other inflammatory biomarkers like TNF-α and IL-10 and diabetic foot ulcers. It was demonstrated that T2DM patients with foot ulcers had lower Apo A1 and IL-10 than T2DM patients without foot ulcers. Low Apo A1 and IL-10 had high sensitivity, specificity, and predictive values in patients with DFU, with IL-10 being a superior biomarker to Apo A1 for the development of DFU. However, none of these showed utility in estimating the disease severity. This could be attributed to fewer patients in the severe grades of ulcer-like grade 4 and 5 and more in grade 3.

It is well known that DFU is a severe complication of diabetes associated with poor quality of life and increased financial burden on the family and hence on the community. Therefore, if certain beacons can serve as early indicators in developing diabetic foot ulcers, this can benefit the patients. The inflammatory state associated with ulcers results in the release of various non-specific cytokines of proinflammatory and anti-inflammatory nature. Multiple factors play a role in the development of foot ulcers. In this study, different variables were compared between DFU patients and patients with diabetes and without foot ulcers to try and establish the association of Apo A1 with the severity of diabetic foot. The overall characteristics of the patient population were similar to various other studies in India as mentioned below and so are generalizable to the population of the subcontinent.

The mean age of the ulcer group was 54.85 ± 9.6 years, and the prevalence in male gender in our study is similar to other studies [1,17,18]. Increasing age and male gender are known independent risk factors for the development of diabetes, as males have a higher exposure to trauma, use improper footwear, and have high foot pressure. Our study has found age to be a prominent risk factor, whereas found gender is not to be a risk factor for DFU. Few studies have found no difference in sex between diabetic patients with and without foot ulcers [19,20]. We assume that the degree of neuropathy, joint mobility, and foot pressure was the same in males and females, and this could be the reason for gender not being a risk factor in this population.

Results of Chi-square and Exact measures of association suggested several factors with potential association with DFU in our study. There was a significant association of obesity (BMI> 25 kg/m^2^) on the risk of developing DFU (OR-3.4, CI: 1.78–6.81), similar to other studies [18,21,22]. Smoking, a longer duration of diabetes (> 10 years), and a family history of diabetes mellitus were strongly associated with foot ulcers. As per many studies, this was identified as a risk factor for neuropathy [18,21,23]. Smokers were 2.56 times (CI: 1.07–6.10) more likely to be associated with diabetic foot ulcers. As the duration of diabetes increases, macrovascular and microvascular complications become more common, which further increase the development of DFU. Retinopathy (OR-3.45, CI: 1.57–7.38) had a strong association with DFU. Poor glycemic control was also associated with DFU cases in this study (p = 0.01). The renal function and lipid profile were more deranged in Group-II than in Group-I. However, upon multivariable analysis, from amongst the above factors, our study drew conclusion only of age (b = 3.072, p = 0.034), smoking (b = 3.017, p = 0.035), retinopathy (b = 1.814, p = 0.02)and CKD (b = 1.638, p = 0.01)as independent associated with DFU.

Due to the inflammatory status in DFU, there was a high concentration of pro-inflammatory mediator TNF-α and lower levels of the anti-inflammatory cytokine, IL-10. Lower levels of ApoA1, an anti-inflammatory molecule were strongly associated with DFU. TNF-α is produced by monocytes, macrophages and Tcells affecting many pathways like adhesion, migration, angiogenesis, and apoptosis. In normal skin wounds, TNF-α is quickly released by the vascular endothelium of the ulcer areas, and it initiates the inflammatory process for wound healing [24]. In DFU, which is a chronic non-healing state, there is prolonged and excessive production of TNF-α by M1 macrophages (classically activated macrophages) that promotes a positive amplification circuit. This can impair the migration of keratinocytes and fibroblasts to the wound site and reduce angiogenesis by adversely affecting endothelial progenitor cells mobilization. The most conspicuous response is lowering the ability of macrophages to phagocytose wound debris and apoptotic neutrophils [25]. The consistent hyperglycemia leads to the accumulation of advanced glycation end products and induces nuclear factor-κB( NF-κB), transcription factors related to cytokines like TNF-α [26]. The M2 cells (alternatively activated macrophages) released during wound healing produce IL-10 that attenuate injury and suppress inflammation by releasing vascular endothelial growth factors [27]. Overall in patients with diabetes, an imbalance towards M1 macrophages increases expression of pro-inflammatory cytokines like TNF-α and decreases IL-10 production (r = −0.224, p = 0.004) by M2 cells, and delays wound healing [28]. Studies in diabetic rats have shown that applying neutralizing TNF-α antibody and TNF-α antagonists have promoted healing of the wounds by increasing the migration capacity of keratinocytes [29]. However, amongst the cytokines only IL-10 (b = −0.37, p = 0.000) and not TNF-α (b = 0.002, p = 0.138) could help in predicting DFU. It can be assumed that IL-10 has a complementary action to TNF-α.

ApoA1 has been shown to exert an anti-inflammatory effect on macrophages, thus inhibiting atherogenesis [30]through various mechanisms like mRNA decay of pro-inflammatory cytokines [31]. Experimental studies in mice have shown that injection of ApoA1 leads to an increase in M2 polarisation markers [IL-10] and a decline in inflammatory markers [32]. Therefore, a lower HDL and ApoA1 level shift the cells away from an M2 macrophage phenotype aggravating the condition and delaying the healing of the DFU. ApoA1 was significantly lowered in DFU patients and served as independent markers for DFU occurrence in diabetic patients (b −0.47, p = 0.012). There was a negative correlation of ApoA1 with TNF-α( r = −0.186, p = 0.018) and a positive correlation with IL-10 (r = 0.310, p = 0.000), which are pro-inflammatory and anti-inflammatory biomarkers. A decreased HDL is associated with a diminished inflammatory immune response. However, although there was no difference in HDL level in our study, the ApoA1 content was lowered in ulcer patients. This could indicate the endogenous anti-inflammatory role of ApoA1. Keeping all these factors into consideration, Apo A1 can be implicated to be an evolving biomarker for the early diagnosis of diabetic foot ulcers.

Smoking was found to be independently associated with DFU in our study population. The oxidative damage due to smoking induces glycotoxins inducing cell damage to vulnerable neural tissues [33]. Cigarette smoking also induces insulin resistance, thereby corroborating the presence of insulin requirements in these patients.

It is notable that early diagnosis and treatment aid in reducing the incidence of DFU, which is expected to reduce other complications like amputation and mortality. Therefore, we propose ApoA1 and IL-10 as inflammatory biomarkers for the early diagnosis of DFU. Expression of serum ApoA1 and IL-10 showed high AUC in the diagnosis of DFU, with IL-10 showing greater accuracy than ApoA1. Even though these two anti-inflammatory markers could not differentiate between the different grades of ulcers, this does not dissuade the combined use of these biomarkers can speculate DFU.

In terms of study limitations, since this is a cross-sectional study, the dynamics of this biomarker can also be well studied with prospective follow-up studies and taking a larger population in each ulcer grade. The study of time-dependent changes in these inflammatory markers during treatment would further reveal more information about the role of ApoA1 and IL-10 in DFU. This study was also carried out in a single tertiary care hospital with a limited representation of the entire Indian population with DFU. However, further studies are required to completely elucidate its role as a biomarker, set target serum levels, and increase its clinical use.

## 5. Conclusion

Although molecular targets like a pro and anti-inflammatory cytokines can serve as biomarkers, it is challenging to pinpoint them for DFU. However, the present study has demonstrated that Apo A1 and IL-10 are significantly reduced in patients with DFU and are correlated with clinical and laboratory indices. Although these biomarkers cannot monitor the severity of DFU, further prospective trials using cut-off values would aid in the early prediction of DFU so that timely management of these patients can be undertaken. Anti-inflammatory molecules, ApoA1 and IL-10, can serve as novel predictors for the occurrence of DFU and thus reduce the risk of morbidity and mortality.

## Supplementary Data

Supplementary Fig. 1 Box plot representation of ApoA1 and IL-10 in patients with DFU1 (n ¼ 20)and DFU2(n ¼ 55).

**Supplementary Table 1:** Wagner’s classification of diabetic ulcer

Ulcer grading	Clinical description
Grade 0	Intact skin (impending ulcer)
Grade 1	Superficial ulcer
Grade 2	Deep ulcer, no bone involvement or abscess
Grade 3	Abscess with bone involvement (osteomyelitis)
Grade 4	Localized gangrene e.g. toes, heel
Grade 5	Extensive gangrene involving entire foot

**Supplementary Table 2:** Correlation matrix of ApoA1 with TNF-α and IL-10

		Apo A1	TNF-α	IL-10
Apo A1	Pearson Correlation	1	−0.186 *	0.310 **
	Sig. (2-tailed)		0.018	0.000
TNF-α	Pearson Correlation	−0.186 *	1	−0.224 **
	Sig. (2-tailed)	0.018		0.004
IL-10	Pearson Correlation	0.310 **	−0.224 **	1
	Sig. (2-tailed)	0.000	0.004	

* Correlation is significant at the 0.05 level (2-tailed).

** Correlation is significant at the 0.01 level (2-tailed).

## Figures and Tables

**Table 1:** Demographic, clinical, and biochemical characteristics in the study groups.

Parameters	Group-I (n = 77)	Group-II (n = 75)	P-value
Age (years)	51.37 ± 10	54.85 ± 9.6	0.03
Male/Female	48 (62.3)/29 (37.4)	59 (78.7)/16 (21.3)	0.02
BMI (kg/m^2^)	22.53 ± 5.05	24.37 ± 4.24	0.01
Duration of diabetes (years)	7.23 ± 5.02	8.78 ± 6.9	0.005
Duration of diabetes; >10 years/<10 years	21 (27.2)/56 (72.7)	32(42.7)/43 (57.3)	0.04
Active smoking	9 (11.7)	19 (25.3)	0.03
Family history of diabetes	21 (27.2)	33 (44)	0.03
HbA1C (%)	8.68 ± 1.86	9.44 ± 1.76	0.01
Creatinine (mg/dL)	1.01 ± 0.23	1.23 ± 0.44	0.00
eGFR (mL/min/1.73m^2^)	80.99 ± 18.54	65.25 ± 20.36	0.00
Uric acid (mg/dL)	5.7 ± 1.37	6.82 ± 2.25	0.00
Cholesterol (mg/dL)	152.8 ± 44.73	191.52 ± 50.62	0.00
Triglycerides (mg/dL)	83.24 ± 32.57	136.47 ± 92.9	0.00
HDL (mg/dL)	44.8 ± 7.61	39.8 ± 5.6	0.002
LDL (mg/dL)	74.57 ± 47.21	122.3 ± 49.46	0.00
Apo A1 (mg/dL)*	97.0 (24.5)	76.0 (31.5)	<0.0001
TNF-α (pg/mL)*	65 (170.53)	193.0 (142.38)	0.0001
IL-10 (pg/mL)*	160.3 (133.33)	32.0 (42.93)	<0.0001

Values are in mean(SD), n(%), median (interquartile range).

**Table 2:** The measure of association of independent variables in the study population.

Independent variable	Group I (n = 77)	Group II (n = 75)	Odds Ratio OR (95% CI)	Risk Ratio	Chi-square test	
	
				RR (95% CI)	Yates	P
BMI >25 kg/m^2^	25 (32.4)	47 (62.6)	3.4 (1.78–6.81)	1.86 (1.32–2.6)	12.71	<0.0002
Hypertension	31 (40.3)	20 (26.7)	0.53 (0.27–1.12)	0.72 (0.48–1.05)	2.5	0.07
Retinopathy	12 (15.6)	29 (8.7)	3.45 (1.57–7.38)	1.70 (1.26–2.29)	9.13	0.001
CKD (eGFR<60 mL/min/1.73m^2^)	6 (7.8)	33 (44)	7.85 (3.19–19.34)	2.2 (1.66–2.9)	22.1	<0.001
Smoking	9 (12)	19 (25)	2.56 (1.07–6.10)	1.50 (1.09–2.07)	3.84	0.03
Family history of diabetes	21 (27)	33 (44)	2.09 (1.06–4.12)	1.42 (1.04–1.94)	3.94	0.03
Total Cholesterol >150 mg/dL	30 (39)	61 (81)	6.82 (3.25–14.3)	2.92 (1.80–4.72)	26.66	0.001
Triglycerides >200 mg/dL	2 (3)	15 (20)	9.37 (2.06–42.6)	1.98 (1.53–2.56)	9.89	0.0005
HDL (<40 mg/dL)	11 (14)	17 (23)	1.75 (0.76–4.05)	1.2 (0.91–1.84)	1.26	0.19
LDL (>100 mg/dL)	10 (13)	15 (20)	1.67 (0.69–4.00)	1.27 (0.87–1.83)	0.89	0.25

Abbreviations: BMI-body mass Index, eGFR-estimated glomerular filtration rate, HDL-high density lipoprotein, LDL-low density lipoprotein, ApoA1-apolipoprotein A1, TNF- tumor necrosis factor, IL-interleukin.

**Table 3:** Multinomial logistic regression analysis in patients of diabetic foot ulcers.

Variables	Likelihood Ratio Tests		Parameter Estimates	
	
	Chi-Square	Significance	Regression coefficients	Significance
Age	4.884	0.027	3.072	0.034
Gender	0.273	0.601	0.381	0.602
BMI	0.076	0.783	0.023	0.784
Smoking	5.321	0.032	3.017	0.035
Duration of diabetes	0.014	0.906	−0.007	0.906
Hypertension	2.40	0.127	−1.128	0.130
Retinopathy	6.136	0.013	1.814	0.020
eGFR	4.390	0.024	1.638	0.010
HbA1C	1.536	0.293	0.764	0.317
Apo A1 (mg/dL)	7.779	0.005	−0.047	0.012
TNF-α (pg/mL)	2.197	0.138	0.002	0.148
IL-10 (pg/mL)	59.73	0.000	−0.037	<0.0001

**Table 4:** Accuracy of ApoA1 and IL-10 in diabetic foot ulcers.

	Cut off	AUC	Sensitivity (%)	Specificity (%)	NPV (%)	PPV (%)	ACC (%)
ApoA1	89.82	0.75 (0.676,0.827)	70.0	65.0	74.0	76.05	75.0
IL-10	78.8	0.93 (0.891,0.970)	88.8	82.5	94.2	85.5	89.5

AUC-Area under the curve, NPV-Negative predictive value, PPV-Positive predictive value, ACC- Accuracy.

## References

[1] Seth A Attri AK Kataria H Kochar S Seth SA Gautam N Clinical profile and outcome in patients of diabetic foot infection Int J App Basic Med Res 2019 9 14 9 10.4103/ijabmr.IJABMR_278_18 PMC6385536 30820414

[2] Musa IR Ahmed MON Sabir EI Alsheneber IF Ibrahim EME Mohamed GB Factors associated with amputation among patients with diabetic foot ulcers in a Saudi population BMC Res Notes 2018 11 260 29703224 10.1186/s13104-018-3372-z PMC5921536

[3] Ugwu E Adeleye O Gezawa I Okpe I Enamino M Ezeani I Predictors of lower extremity amputation in patients with diabetic foot ulcer: findings from MEDFUN, a multi-center observational study J Foot Ankle Res 2019 12 34 31223342 10.1186/s13047-019-0345-y PMC6570910

[4] Raghav A Khan ZA Labala RK Ahmad J Noor S Mishra BK Financial burden of diabetic foot ulcers to the world: a progressive topic to discuss always TherAdvEndocrinolMetab 2018 9 1 29 31 10.1177/2042018817744513 PMC5761954 29344337

[5] Patel S Maheswari A Chandra A Biomarkers for wound healing and their evaluation J Wound Care 2016 25 1 46 55 26762498 10.12968/jowc.2016.25.1.46

[6] Morey M Gaora PO Pandit A Helary C Hyperglycemia acts in synergy with hypoxia to maintain the pro-inflammatory phenotype of macrophages PLoS ONE 2019 14 8 e0220577 31415598 10.1371/journal.pone.0220577 PMC6695165

[7] DeClue CE Shornick LP The cytokine milieu of diabetic wounds Diabetes Manag 2015 5 6 525 37

[8] Steen EH Wang X Balaji S Butte MJ Bollyky PL Keswani S The role of anti-inflammatory cytokine interleukin-10 in tissue fibrosis Advances in wound care 2019 9 4 184 98 10.1089/wound.2019.1032 PMC7047112 32117582

[9] Miao M Miao M Niu Y Xie T Yuan B Qing C Diabetes- impaired wound healing and altered macrophage activation: a possible pathophysiologic correlation Wound Repair Regen 2012 20 203 13 22380690 10.1111/j.1524-475X.2012.00772.x

[10] Mangaraj M Nanda R Panda S Apolipoprotein A-I: A molecule of diverse function Indian J ClinBiochem 2016 31 3 253 9 10.1007/s12291-015-0513-1 PMC4910842 27382195

[11] Georgila K Vyrla D Drakos E Apolipoprotein A-I (ApoA-I), immunity, inflammation and cancer Cancers 2019 11 8 1097 10.3390/cancers11081097 PMC6721368 31374929

[12] Doni A D’Amico G Morone D Mantovani A Garlanda C Humoral innate immunity at the crossroad between microbe and matrix recognition: the role of PTX3 in tissue damage Semin Cell Dev Biol 2017 61 31 40 27476448 10.1016/j.semcdb.2016.07.026 PMC5419421

[13] Tesfaye S Boulton AJ Dyck PJ Freeman R Horowitz M Kempler P Toronto Diabetic Neuropathy Expert Group Diabetic neuropathies: update on definitions, diagnosis criteria, estimation of severity, and treatments Diabetes Care 2010 33 2285 93 20876709 10.2337/dc10-1303 PMC2945176

[14] Frykberg RG Diabetic foot ulcers: pathogenesis and management Am Fam Physician 2002 66 1655 63 12449264

[15] Passarella P Kiseleva TA Valeeva FV Gosmanov AR Hypertension management in diabetes:2018 update Diabetes Spectrum 2018 31 3 218 24 30140137 10.2337/ds17-0085 PMC6092891

[16] Vijayaraghavan K Treatment of dyslipidemia in patients with type 2 diabetes Lipida Health Dis 2010 9 144 10.1186/1476-511X-9-144 PMC3022752 21172030

[17] Kateel R Augustine AJ Prabhu S Ulll S Pai M Adhikari P Clinical and microbiological profile of diabetic foot ulcers in a tertiary care hospital Diab Met Syndr: Clin Res Rev 2017 12 1 27 30 10.1016/j.dsx.2017.08.008 28867530

[18] Vibha SP Kulkarni MM Ballala ABK Kamat A Maiya GA Community based study to assess the prevalence of diabetic foot syndrome and associated risk factors among people with diabetes mellitus BMC Endocrine Disorders 2018 18 43 29940924 10.1186/s12902-018-0270-2 PMC6020220

[19] Jbour AS Jarrah NS Radaideh AM Prevalence and predictors of diabetic foot syndrome in type 2 diabetes mellitus in Jordan Saudi Med J 2003 24 761 4 12883610

[20] Al Kafrawy NA Mustafa EA Dawood ADA Ebaid OM Ahmed Zidane OM Study of risk factors of diabetic foot ulcers Menoufia Med J 2014 27 28 34

[21] Nongmaithem M Bawa APS Pithwa AK Bhatia SK Singh G Gooptu S A study of risk factors and foot care behavior among diabetics J Family Med Prim Care 2016 5 399 27843849 10.4103/2249-4863.192340 PMC5084569

[22] Jaiswal M Lauer A Martin CL Bell RA Divers J Dabelea D Peripheral neuropathy in adolescents and young adults with type 1 and type 2 diabetes from the SEARCH for diabetes in youth follow-up cohort Diabetes Care 2013 36 3903 8 24144652 10.2337/dc13-1213 PMC3836139

[23] Shahi SK Kumar A Kumar S Singh SK Gupta SK Singh TB Prevalence of diabetic foot ulcer and associated risk factors in diabetic patients from North India Jour Diab Foot Compl 2012 4 3 83 91

[24] Birkl D Quiros M Garcia-Hernandez V Zhou DW Brazil JC Hilgath R TNF alpha promotes mucosal wound repair through enhance platelet activating factor receptor signalling in the pithelium Mucosal Immunol 2019 12 4 900 18 10.1038/s41385-019-0150-8 PMC6599476 30971752

[25] Ganesh GV Ramkumar KM Macrophages mediation in normal and diabetic wound healing responses Inflamm Res 2019 69 347 63 10.1007/s00011-020-01328-y 32146517

[26] Qing C The molecular biology in wound healing and non-healing wound Chin J Traumatol 2017 20 189 93 28712679 10.1016/j.cjtee.2017.06.001 PMC5555286

[27] Xu F Zhang C Graves DT Abnormal cell responses and role of TNF-α in impaired diabetic wound healing Biomed Res Int 2013 2013 754802 23484152 10.1155/2013/754802 PMC3581278

[28] Hendrijantini N Sitalaksmi RM Ari MDA Hidayat TJ Putri Pan Sukandar D The expression of TNF-α, IL-1β and IL-10 in the diabetes mellitus condition induced by the combination of spirulina and chitosan Bali Med J 2020 9 1 22 6

[29] Raziyeva K Kim Y Zharkinbekov Z Kassymbek K Jimi S Saparov A Immunology of acute and chronic wound healing Biomolecules 2021 11 5 700 10.3390/biom11050700 34066746 PMC8150999

[30] Umemoto T Han CY Mitra P Averill MM Tang C Goodspeed L Apolipoprotein A1 and high-density lipoprotein have anti-inflammatory effects on adipocytes via cholesterol transporters Circ Res 2013 112 10 1345 54 23501697 10.1161/CIRCRESAHA.111.300581 PMC3767575

[31] Yin K Deng X Mo ZC Zhao GJ Jiang J Cui LB Tristetraprolin-dependent post-transcriptional regulation of inflammatory cytokine mRNA expression by Apolipoprotein A1 Role of ATP binding membrane cassette transporter A1 and signal transducer and activator of transcription3 J Biol Chem 2011 286 13834 45 21339300 10.1074/jbc.M110.202275 PMC3077584

[32] Medbury HJ Williams H Li S Fletcher JP The bidirectional relationship between cholesterol and macrophage polarization J Clin Cell Immunol 2015 6 303

[33] Xia N Morteza A Yang F Cao H Wang A Review of the role of cigarette smoking in diabetic foot J Diabetes Investig 2019 10 202 15 10.1111/jdi.12952 PMC6400172 30300476

