# Supplementary figures and images for: A study of apolipoprotein A1(ApoA1) and interleukin-10(IL-10) in diabetes with foot ulcers
Source: Biomedicine (Taipei). 2022 Mar 1;12(1):30–8. doi: 10.37796/2211-8039.1279 (PMC9236711; doi:10.37796/2211-8039.1279)

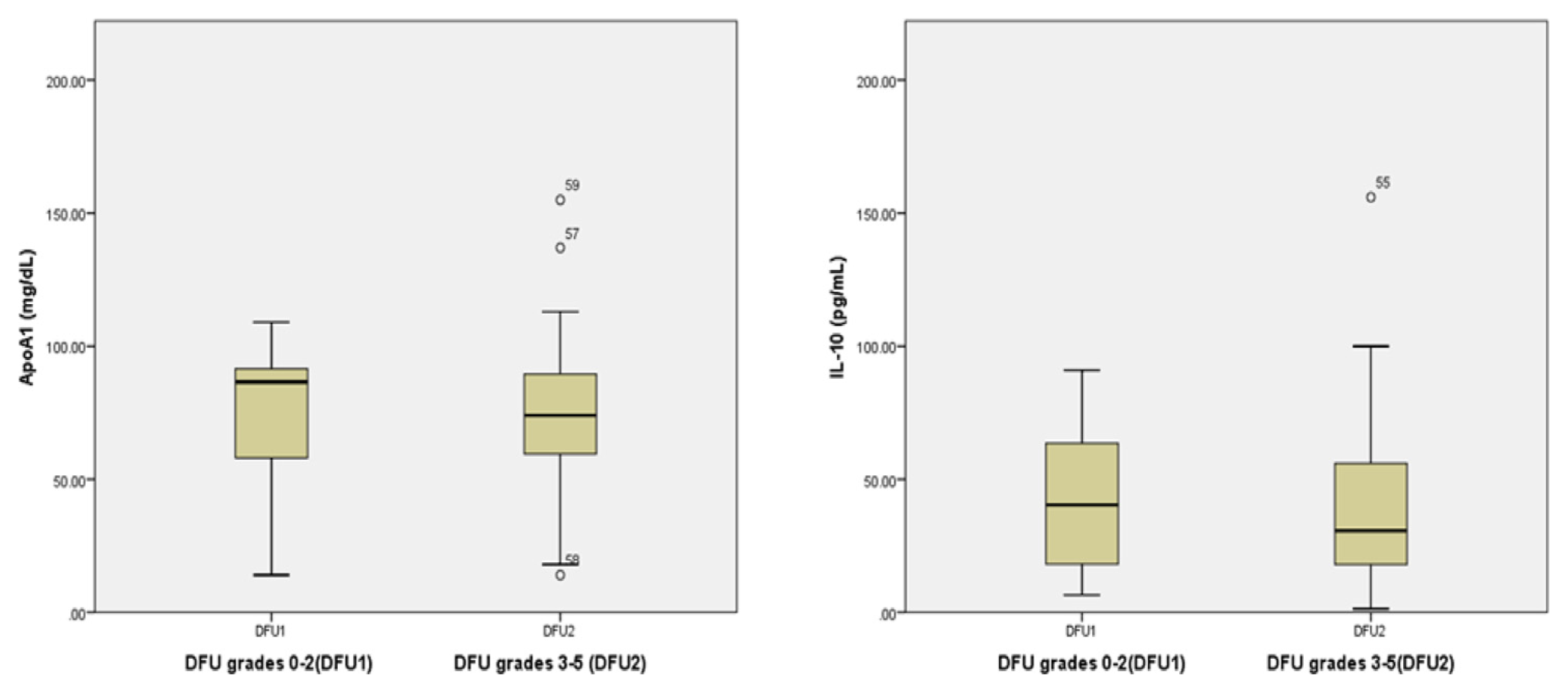

Supplement: Supplementary Fig. 1 — Box plot representation of ApoA1 and IL-10 in patients with DFU1 (n ¼ 20)and DFU2(n ¼ 55). [file bmed-12-01-030s1.tif]
